# Host species composition influences infection severity among amphibians in the absence of spillover transmission

**DOI:** 10.1002/ece3.1385

**Published:** 2015-03-05

**Authors:** Barbara A Han, Jacob L Kerby, Catherine L Searle, Andrew Storfer, Andrew R Blaustein

**Affiliations:** 1 Cary Institute of Ecosystem StudiesMillbrook, New York, 12545; 2 Biology Department, University of South Dakota414 E. Clark St., Vermillion, South Dakota, 57069; 3 Department of Ecology and Evolutionary Biology, University of MichiganAnn Arbor, Michigan, 48109; 4 School of Biological Sciences, Washington State UniversityPullman, Washington, 99164; 5 Department of Zoology, Oregon State University3029 Cordley Hall, Corvallis, Oregon, 97331

**Keywords:** *Batrachochytrium*, biodiversity, chytridiomycosis, community structure, dilution effect, disease risk, diversity-disease, tadpole

## Abstract

Wildlife epidemiological outcomes can depend strongly on the composition of an ecological community, particularly when multiple host species are affected by the same pathogen. However, the relationship between host species richness and disease risk can vary with community context and with the degree of spillover transmission that occurs among co-occurring host species. We examined the degree to which host species composition influences infection by *Batrachochytrium dendrobatidis* (Bd), a widespread fungal pathogen associated with amphibian population declines around the world, and whether transmission occurs from one highly susceptible host species to other co-occurring host species. By manipulating larval assemblages of three sympatric amphibian species in the laboratory, we characterized the relationship between host species richness and infection severity, whether infection mediates growth and survivorship differently across various combinations of host species, and whether Bd is transmitted from experimentally inoculated tadpoles to uninfected tadpoles. We found evidence of a dilution effect where Bd infection severity was dramatically reduced in the most susceptible of the three host species (*Anaxyrus boreas*). Infection also mediated survival and growth of all three host species such that the presence of multiple host species had both positive (e.g., infection reduction) and negative (e.g., mortality) effects on focal species. However, we found no evidence that Bd infection is transmitted by this species. While these results demonstrate that host species richness as well as species identity underpin infection dynamics in this system, dilution is not the product of reduced transmission via fewer infectious individuals of a susceptible host species. We discuss various mechanisms, including encounter reduction and antagonistic interactions such as competition and opportunistic cannibalism that may act in concert to mediate patterns of infection severity, growth, and mortality observed in multihost communities.

## Introduction

The relationship between biodiversity and infectious diseases is complex. On the one hand, infectious agents are increasingly appreciated for their role in influencing community dynamics, particularly through affecting ecological interactions among coexisting hosts. For example, pathogens and parasites (considered synonymous herein) can alter social (Altizer et al. 2003; Behringer et al. 2006; Han et al. 2008), competitive (Park 1948; Kiesecker and Blaustein 1999; Borer et al. 2007), or predator–prey interactions (Joly and Messier 2004; Parris and Beaudoin 2004; Han et al. 2011). Conversely, community dynamics can have large effects on infectious disease dynamics. Recent debate about the role of biodiversity in disease risk has surrounded the commonality of the *dilution effect*, a negative relationship between biodiversity and disease risk. While the dilution effect has been observed in a number of studies, the opposite pattern is sometimes found (a positive relationship between biodiversity and disease risk, termed the *amplification effect*) (reviewed in Keesing et al. 2010). In fact, a number of studies investigating the dilution effect (Ostfeld and Keesing 2013; Salkeld et al. 2013; Wood and Lafferty 2013) have found that effects of biodiversity on infectious diseases can be nuanced and complex.

There are a number of mechanisms that can lead to a positive or negative relationship between biodiversity and disease. For example, generalist pathogens with a broad host range may persist and amplify more easily in communities where there is higher richness of suitable species to infect (Hatcher et al. 2006; Keesing et al. 2010). Conversely, infection could be diluted in species-rich communities due to the presence of less suitable alternative host species, or when multiple coexisting species ensure that no single species reaches densities high enough to support epidemic disease. Dilution could also occur if adding species directly reduces the probability of acquiring infection (*transmission reduction*; Keesing et al. 2006), or lead to a decoy effect where host species become infected but do not propagate new infection (Johnson and Thieltges 2010). There are now multiple empirical examples supporting several of these mechanisms leading to disease dilution and amplification (reviewed in Keesing et al. 2006, 2010), suggesting that both the richness and abundance of key host species influence community-level patterns of infection. Thus, whether dilution or amplification effects occur will greatly depend on species composition, individual species density, the degree of spillover infection from particularly competent host species, and the order in which species are lost or gained in a system as biodiversity is altered (Ostfeld and LoGiudice 2003).

We investigated the effects of species composition on disease dynamics using a multihost–pathogen system involving a fungal chytrid pathogen (*Batrachochytrium dendrobatidis*, hereafter Bd) and three amphibian host species. This recently emerged amphibian fungal pathogen affords an ideal opportunity to examine how host species composition influences the severity of shared infection. Bd infects numerous amphibian species worldwide (Fisher et al. 2009; Olson et al. 2013) and is prominently associated with amphibian population declines in several regions (Kilpatrick et al. 2009). Species vary in susceptibility to Bd (Blaustein et al. 2005; Searle et al. 2011b), and it is likely that species community composition plays a role in mitigating or exacerbating disease in various communities. For example, two previous studies have demonstrated a negative relationship between amphibian host diversity and Bd infection (Searle et al. 2011a; Venesky et al. 2013), with one study attributing this relationship to the removal of infectious zoospores in the water column by obligate filter-feeding tadpole hosts (Venesky et al. 2013). More generally, in multihost systems, interspecific interactions (including parasite consumption, direct competition, and opportunistic cannibalism) may interact with differential host susceptibility to influence infection patterns.

In this study, we experimentally manipulated species composition while holding density constant to determine the effects of species richness on infection and other host life-history characteristics. We demonstrate that the dilution effect does occur in this system on the most susceptible of the three host species, but not as a general function of richness. We also show that species composition can strongly influence host growth and mortality, highlighting the importance of interspecific interactions in driving infection patterns and host life history in this system.

## Materials and Methods

### Study system

We investigated three amphibian host species that predominate montane communities of the Oregon Cascades, which co-occur naturally, and have been found positively infected by Bd at larval and postmetamorphic life stages: Western toads (*Anaxyrus boreas*), Cascades frogs (*Rana cascadae*), and Pacific tree frogs (*Pseudacris regilla*; hereafter *Anaxyrus*, *Rana*, and *Pseudacris*, respectively). We manipulated combinations of these three amphibian species to investigate how Bd infection severity, growth, and survivorship of each species are influenced by species richness. We examined infection severity in single-, two-, and three-species combinations for all species and determined whether Bd mediates growth and mortality in hosts among various species combinations.

### Experimental design

All tadpoles were reared in the laboratory from eggs collected from montane water bodies of the Oregon Cascades (U.S.A.). Upon reaching the free-swimming stage in the laboratory (stages 26–27; Gosner 1960), tadpoles were assigned to one of two pathogen treatments (exposed [Bd+] or unexposed [Bd−]) and one of seven species combinations (single-species groups, *Pseudacris* [P]*, Anaxyrus* [A], and *Rana* [R]; two-species combinations [PA, AR, PR]; and all three species together [PAR]). Each combination was initiated with six tadpoles per experimental unit such that the total number of hosts was identical and only species composition varied across treatments. This 7 × 2 design was replicated eight times for a total of 112 experimental containers (30 × 17 × 9 cm). Bd+ treatments were given an exposure dose of 4.38 × 10^8^ zoospores/container one time at the beginning of the experimental period (Appendix S1). Animals were fed a 2:1 mixture of ground alfalfa pellets and fish flakes (Tetramin).

Containers were observed daily for 40 days during which all animals remained in the free-swimming stages (stages 26–30; Gosner 1960). Mortality was allowed to occur without replacement. Tadpole carcasses were removed during daily observations and preserved in 70% ethanol using sterile forceps for real-time PCR quantification of Bd loads in host tissue. In addition, after 40 days, all remaining tadpoles were euthanized and preserved in 70% ethanol for real-time PCR. We recorded both mass and body length for each tadpole prior to PCR.

Infection severity was quantified in Bd-specific genome equivalents (GE) using a variation on established real-time quantitative PCR methods (Boyle et al. 2004; details in Appendix S1). Values obtained from the real-time PCR reaction are mean Bd zoospore genome equivalents per nanogram of excised mouthpart tissue. This method allows quantification of Bd infection while accounting for potential differences in body size among treatments and species. Additionally, we conducted a trial to detect intra- and interspecific transmission of Bd from infected *Anaxyrus* tadpoles to uninfected conspecifics and uninfected *Pseudacris* tadpoles, respectively. See Appendix S1 for further details on animal collection, husbandry, inoculation, and q-PCR techniques.

### Statistical analyses

We conducted statistical analyses to examine Bd infection severity, growth, and mortality among various host species combinations. Analyses of infection severity were conducted on a subset of the data (those that were inoculated with Bd), and analyses of how Bd and species composition influenced growth and mortality were conducted on the full dataset (Bd+ and control treatments). Separate analyses were conducted for each focal species. We did not perform a single global analysis on the effects of species combination as the experimental design was necessarily nonorthogonal (i.e., not all species were represented in all combinations). All analyses were conducted in R (R Core Team 2014).

Overall differences in Bd infection severity between the three host species were examined using a generalized linear model (GLM) with identity link function (mean log(Bd) ∽ focal species + mean day of death. To examine whether Bd infection severity was influenced by species combinations, we used the following GLM model for each species: mean log(Bd) ∽ species combination + mean day of death, gaussian (link = identity). We also examined the severity of infection more generally by asking whether mean Bd infection differs across 1, 2, 3 species combinations using a Kruskal–Wallis *χ*^2^ test.

We also examined growth (mass and length) for each host species using GLMs with identity link functions. Covariates for this model included Bd treatment, species combination, Bd*combo interaction, and mean day of death. We examined mortality using log-linear models, including Bd treatment, species combination, and their interaction as covariates.

### Bd transmission trials

We conducted a trial to determine whether *Anaxyrus* tadpoles can transmit Bd infection to other tadpoles. We focused on Anaxyrus because they are susceptible to severe infection with heavy pathogen loads. To determine whether spillover transmission can occur from this species, we used identical inoculation and diagnostic methods as in the main experiment and combined two infected *Anaxyrus* tadpoles with uninfected tadpoles in three treatments: (1) Two uninfected *Anaxyrus* tadpoles (*N* = 3), (2) 12 uninfected *Anaxyrus* tadpoles, and (3) uninfected *Anaxyrus* and *Pseudacris* tadpoles (three of each, *N* = 2) in experimental containers. In all treatments, infected *A. boreas* tadpoles interacted freely with uninfected tadpoles and were differentiated from originally uninfected animals using tail notches (a semicircular biopsy taken from the dorsal fin using a 2-mm biopsy punch). Notches were still visible at the end of the experiment (40 days). Bd infection was quantified using real-time PCR.

## Results

### Infection severity

**Figure 1:**
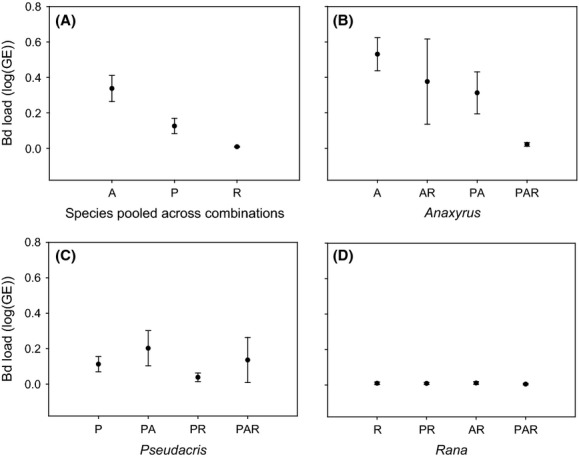
Infection severity measured in mean log (genome equivalents) for three amphibian species pooled across all species combinations (A) and specifically for *Anaxyrus boreas* (B), *Pseudacris regilla* (C) and *Rana cascadae* (D) within their assigned species combinations.

Real-time q-PCR analyses of tadpole mouthparts (*N* = 324) confirmed that inoculation was sufficient to cause Bd infection in all species and all species combinations. All tadpoles tested from control treatments were negative for Bd. Overall, mean infection severity differed among species. *Anaxyrus* had the highest Bd loads, *Pseudacris* showed intermediate loads, and *Rana* had low or undetectable Bd loads (Fig.1A, Table S1).

Infection severity differed in focal species across various host species combinations. In *Anaxyrus*, infection was most severe in *Anaxyrus-*only treatments (A) and less severe when combined with either *Pseudacris* (PA) or with both *Pseudacris* and *Rana* (PAR) (Fig.1B, Table S1). A significant “day” effect indicated reduced infection among *Anaxyrus* tadpoles that survived longer (Table S1). There was no effect of species combination or day on infection severity in *Pseudacris* or *Rana* (Table S1, Fig.1C and D). Overall, infection severity (i.e., all Bd+ species considered together) did not differ across the three richness treatments (single, two, and three species combinations; Kruskal–Wallis *χ*^2^ test; H = 4.90, df 2, *P* = 0.09).

### Growth

**Figure 2:**
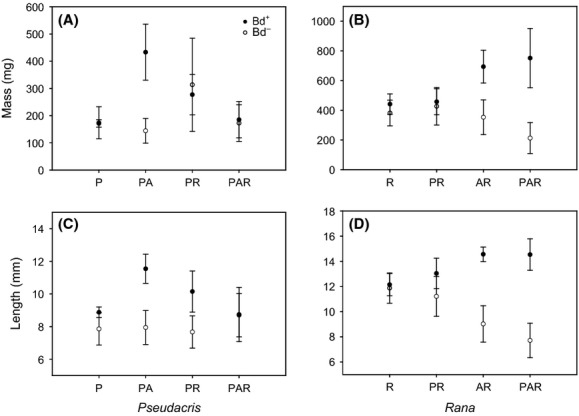
Mean mass (mg) and length (mm) of tadpoles in Bd+ (•) and Bd− treatments (○) for *Pseudacris regilla* (A, C) and *Rana cascadae* (B, D) across species combinations.

Mass and length analyses were only conducted on *Pseudacris* and *Rana*, as high mortality and partial predation of soft tissues (intestines, tails) prevented the collection of adequate length and mass data for *Anaxyrus* (particularly in Bd+ AR and PAR combinations). Bd infection in tadpoles is localized in keratinized mouthparts and toothrows which remained intact following mortality for uncompromised q-PCR quantification of Bd severity for the majority of tadpoles (*N* = 543), but there were 105 individual tadpoles whose bodies went missing during the experiment (Table S2). *Pseudacris* increased in length when combined with *Anaxyrus* in Bd+ compared with Bd— PA combinations (Fig.2A and C, Table S3). Similarly, Bd+ *Rana* in AR and PAR combinations grew larger and longer compared with the *Rana* in Bd− treatments (Fig.2B and D, Table S3). There was no effect of “day” for either species.

### Mortality

Species combination affected *Anaxyrus* and *Pseudacris* mortality (Table1). *Anaxyrus* mortality was highest when combined with *Rana*, *Pseudacris* mortality was lowest when combined with *Anaxyrus*, and *Rana* mortality was lowest in Bd+ treatments (Table1, Fig. S1). *Pseudacris* and *Rana* mortality were lowest in the Bd+ treatments containing *Anaxyrus* (Fig. S1B and D).

**Table 1:** Log-linear tests of the mean proportion mortality observed in each host species. Models included infection (Bd) and species combination (combo) as main effects, and a two-way interaction term (Bd:combo). Bolded numbers are statistically significant at α = 0.05

Proportion	*Anaxyrus*			*Pseudacris*			*Rana*		
	DF	*χ*^2^	p	DF	*χ*^2^	*p*	DF	*χ*^2^	*p*
Bd	1	0	1	1	0.6	0.44	1	4.47	**0.03**
Combination	3	13.93	**<0.01**	3	9.51	**0.023**	3	0.85	0.84
Bd*Combo	3	4.86	0.18	3	3.07	0.38	3	7.03	0.07

### Transmission

At the end of the trial, the mean Bd load in the originally infected *Anaxyrus* tadpoles was 31.1 GE (±14.9 SE) and real-time PCR analyses showed that none of the initially uninfected *Anaxyrus* or *Pseudacris* tadpoles became infected by Bd.

## Discussion

Here, we report experimental evidence that species composition and species identity can underlie patterns of infection severity in a multihost system. Specifically, we demonstrated a dilution effect in one species where Bd infection severity in *Anaxyrus* was dramatically reduced in the presence of two additional sympatric host species. However, this pattern was not found in other species; there was no effect of species combination on Bd infection in the other two species (*Pseudacris* or *Rana*), and infection severity did not decrease as a general function of species richness. Instead, the relationship between host species richness and infection severity varied depending on the focal species. These results suggest that dilution effects in studies of natural systems may be missed if infection is examined in one or a few focal species in the community.

Overall, species varied widely in their susceptibility to infection (Fig.1A). Infection loads were highest in *Anaxyrus*, lowest in *Rana*, and intermediate in *Pseudacris*. *Anaxyrus* experienced the most severe infection when in single-species groups (Fig.1B), but treatments combining them with *Pseudacris* and *Rana* reduced infection by nearly an order of magnitude (from 0.6 to near zero GE). In contrast, we observed a trend where *Pseudacris* harbored higher Bd loads when combined with *Anaxyrus*, and lower Bd loads when combined with *Rana* (Fig.1C, PA vs. PR). Although not statistically significant, this trend highlights the possibility that Bd severity in *Pseudacris* may be influenced by spillover infection from highly susceptible *Anaxyrus* such that more *Anaxyrus* tadpoles leads to more severe infections in surrounding hosts. Exploring this possibility through simple transmission trials, we found that while *Anaxyrus* is susceptible to heavy infection by free-swimming Bd zoospores, once infected these tadpoles did not transmit detectable infections to *Pseudacris* or other *Anaxyrus*, even when held in close proximity over a prolonged time period. We therefore conclude that the patterns of Bd severity we observed in *Pseudacris* were unlikely to be caused by spillover transmission from *Anaxyrus* tadpoles. Given that the presence of other host species reduced infection so dramatically in *Anaxyrus* tadpoles, we also considered the possibility that *Pseudacris* and *Rana* were acting as “decoy” hosts for *Anaxyrus*, “soaking up” free-swimming zoospores and acquiring heavy infections and thereby lowering the zoospores available in the water to infect *Anaxyrus*. While we did observe a trend of increasing infection severity in *Pseudacris* when combined with *Anaxyrus*, this trend was not statistically significant. For *Rana*, Bd loads remained consistently low (near zero) regardless of species combination. Thus, it is unlikely that *Rana* was acting as a decoy host for *Anaxyrus*, but it is worth further investigating this possibility for *Pseudacris*.

Another possibility is that some species, such as *Rana* or *Pseudacris* in our study, lower the risk of infection for more susceptible species (like *Anaxyrus*) by more directly reducing the concentration of infectious propagules in the water. In the amphibian-Bd system, this can occur via filter feeding (Altig et al. 2007). Filter-feeding organisms such as amphibians (Venesky et al. 2013) and aquatic zooplankton (Buck et al. 2011; Searle et al. 2013) consume free-swimming Bd zoospores and can reduce infection in larval amphibian hosts. Thus, the consumption of Bd by either *Rana* or *Pseudacris* could have reduced infection for *Anaxyrus*. Feeding behaviours resulting in the direct consumption of zoospores could lower infection risk at the community level through *encounter reduction* (Keesing et al. 2006), where infection is diluted among the more susceptible host species because they encounter fewer infectious propagules in their environment. This “vacuum cleaner effect” may also scale allometrically within and between host species as larger tadpoles consume proportionately more zoospores compared with smaller tadpoles. Thus, as *Pseudacris* and *Rana* were (on average) larger than *Anaxyrus*, their body size may have allowed them to act as diluters through higher rates of Bd consumption.

We observed large differences in growth and mortality among some treatments that we attribute to agonistic interactions that are common in amphibian assemblages. For example, host development and survivorship can be affected by stress associated with predation (Parris and Beaudoin 2004) and competition (Parris and Cornelius 2004). In our study, *Rana* can grow to be much larger than *Pseudacris* and *Anaxyrus* and was often observed nibbling on moribund individuals, which may partially explain the greater mortality rates observed in species combined with *Rana*. Agonistic interactions may also explain why *Anaxyrus* mortality increased while Bd loads decreased to near zero in treatments combining them with other species (Table1, Fig.1, Fig. S1) – although *Anaxyrus* had less severe infections, in the presence of both Bd and another host species they also showed poor survival. Concurrently, *Pseudacris* and *Rana* increased dramatically in size when combined with *Anaxyrus* and Bd (Fig.2, Table S2). Taken together, these patterns could be the result of competitive release following high *Anaxyrus* mortality from both infection and interspecific competition working together in mixed species combinations. Predation of dead or moribund *Anaxyrus* by heterospecific tadpoles may explain the increased growth of *Pseudacris* and *Rana* as well as the paucity of intact *Anaxyrus* guts and tails available for mass and length measurements in our study.

Our study suggests that dilution effects in some multihost–pathogen systems may be driven by key species and host species interactions. For example, the presence of additional host species reduced Bd severity for the more susceptible *Anaxyrus* hosts, highlighting a potentially positive role for high species richness to reduce disease burden. We observed a clear dilution effect when we measured infection severity in only a single species (*Anaxyrus*), but not when focusing on the other two species, nor when we conducted a more general analysis of infection severity across three levels of increasing species richness. Thus, species identity is a key driver of the diversity–disease relationship in our system. However, observing a dilution effect in this multihost system will depend on whether infection is being examined across all host species or in one focal species in the community. Whether the dilution effect that we observed confers any benefit to *Anaxyrus* tadpoles in natural settings is unclear, as the effects of interspecific competition and predation have more immediate impacts on survival than Bd infection at this developmental stage.

Future studies that more explicitly address the dynamic relationship between infection and species interactions will be critical for informing conservation and management efforts, especially in light of the prominent role of Bd infection on amphibian population declines. An open question is how species composition is contributing to the wide variation in Bd severity among populations, which range from disease-related population crashes to low-level infection without obvious mortality events. Graduating up from experimentally tractable multispecies amphibian studies to examine infection dynamics in more natural settings (e.g., setting where tadpoles are allowed to form schools, partition the water column during foraging, and interact with naturally occurring predators) will bring us closer to a mechanistic understanding of the relationships between biodiversity and community disease burden, and how the interactions between species are mediated by infection to influence disease ecology in this system.

## Supporting Information

**Figure S1.** The mean proportion of tadpoles of *Anaxyrus boreas* (A), *Pseudacris regilla* (B), and *Rana cascadae* (C) that died in each of four species combinations and two Bd pathogen treatments.

**Table S1.** Generalized linear models of *Batrachochytrium* infection severity for each of three host species: *Anaxyrus boreas* (Anaxyrus), *Pseudacris regilla* (Pseudacris), and *Rana cascadae* (Rana). Factors for each model include species combinations (Combinations, 4 levels), and day of death (Day).

**Table S2.** The number of missing tadpoles for each of three host species *Anaxyrus boreas* (A), *Pseudacris regilla* (P), and *Rana cascadae* (R) across 7 species combinations and two pathogen treatments (Bd).

**Table S3.** Generalized linear models for mean growth in mass (mg) and length (mm) in tadpoles of two amphibian host species among four species combinations (Combo) and two pathogen treatments (Bd). Models include a two-way interaction term (Bd*combo) and Day as a covariate.
