# Supplementary figures and images for: Host species composition influences infection severity among amphibians in the absence of spillover transmission
Source: Ecol Evol. 2015 Mar 5;5(7):1432–9. doi: 10.1002/ece3.1385 (PMC4395173; doi:10.1002/ece3.1385)

Figure S1

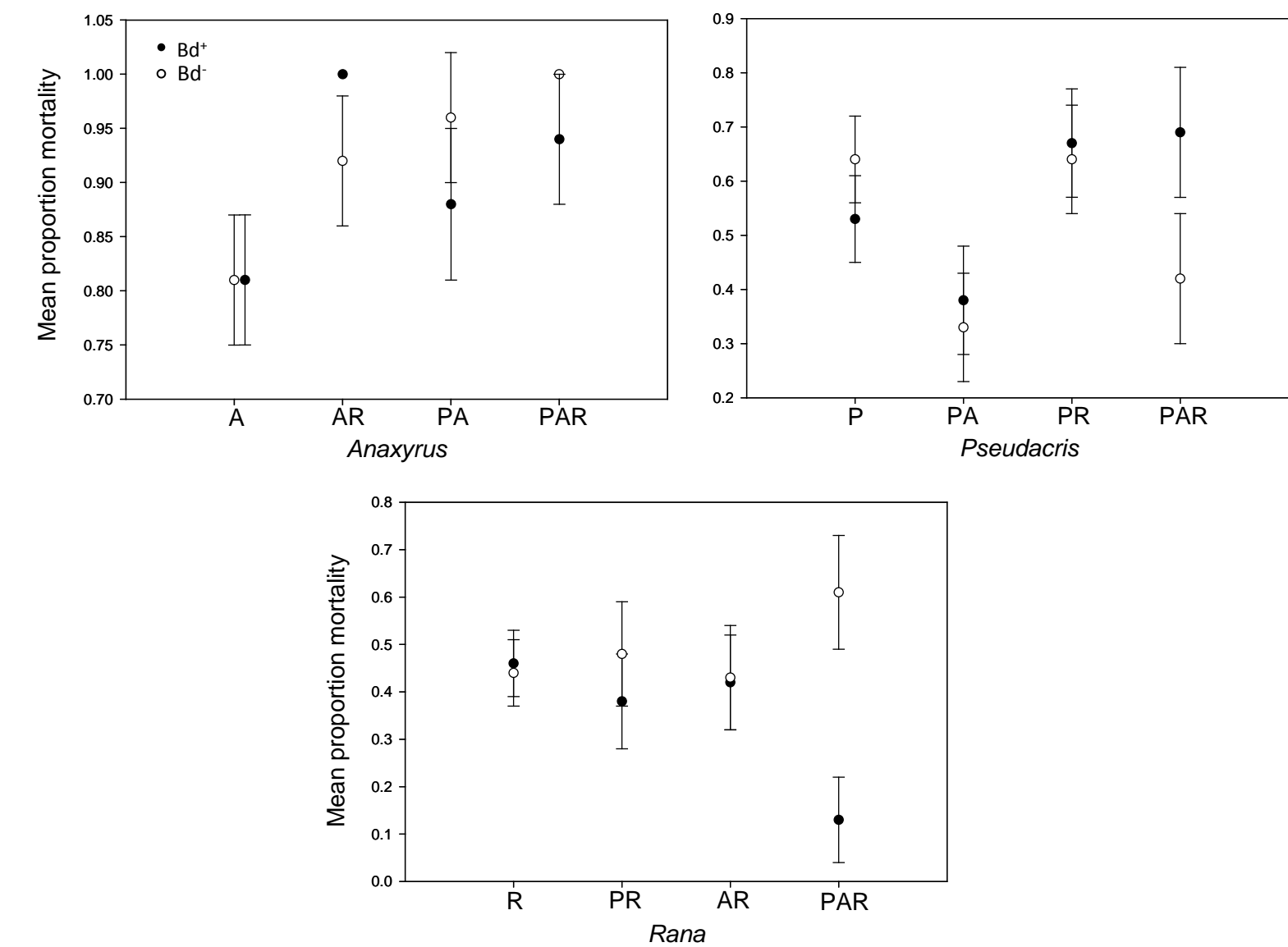

Supplement: Supplementary file 1 — Figure S1. The mean proportion of tadpoles of Anaxyrus boreas (A), Pseudacris regilla (B), and Rana cascadae (C) that died in each of four species combinations and two Bd pathogen treatments. [file ece30005-1432-sd1.pdf]
