# Supplementary material for: Host species composition influences infection severity among amphibians in the absence of spillover transmission
Source: Ecol Evol. 2015 Mar 5;5(7):1432–9. doi: 10.1002/ece3.1385 (PMC4395173; doi:10.1002/ece3.1385)
Supplement: Supplementary file 2 — Table S1. Generalized linear models of Batrachochytrium infection severity for each of three host species: Anaxyrus boreas (Anaxyrus), Pseudacris regilla (Pseudacris), and Rana cascadae (Rana). Factors for each model include species combinations (Combinations, 4 levels), and day of death (Day). [file ece30005-1432-sd2.docx]

|  | **Table S1** |  |  |  |  |  |
| --- | --- | --- | --- | --- | --- | --- |
|  |  |  | estimate | *se* | *t* | *p* |
| Overall | Species | A | 0.40 | 0.06 | 6.17 | **<0.001** |
|  |  | P | -0.15 | 0.08 | -1.79 | 0.07 |
|  |  | R | -0.24 | 0.09 | -2.61 | **0.01** |
|  | Day |  | -4.90E-03 | 3.30E-03 | -1.48 | 0.14 |
| *Anaxyrus* | Combinations: | A | 0.97 | 0.18 | 5.51 | **<0.001** |
|  |  | AR | -0.26 | 0.17 | -1.57 | 0.132 |
|  |  | PA | -0.38 | 0.16 | -2.35 | **0.028** |
|  |  | PAR | -0.57 | 0.18 | -3.26 | **0.004** |
|  | Day |  | -0.03 | 0.01 | -3.16 | **0.004** |
| *Pseudacris* | Combinations: | P | 0.11 | 0.21 | 0.54 | 0.59 |
|  |  | PA | 0.09 | 0.14 | 0.69 | 0.50 |
|  |  | PR | -0.07 | 0.13 | 0.19 | 0.85 |
|  |  | PAR | 0.03 | 0.14 | -0.53 | 0.60 |
|  | Day |  | -6.6E-05 | 0.01 | -0.01 | 0.99 |
| *Rana* | Combinations: | R | 2.5E-03 | 0.01 | -0.27 | 0.79 |
|  |  | AR | 0.02 | 0.01 | 1.72 | 0.10 |
|  |  | PR | 1.1E-03 | 0.01 | -0.12 | 0.91 |
|  |  | PAR | 2.9E-03 | 0.01 | -0.28 | 0.78 |
|  | Day |  | -4.4E-04 | 4.2E-04 | -1.04 | 0.31 |
