# Supplementary material for: Host species composition influences infection severity among amphibians in the absence of spillover transmission
Source: Ecol Evol. 2015 Mar 5;5(7):1432–9. doi: 10.1002/ece3.1385 (PMC4395173; doi:10.1002/ece3.1385)
Supplement: Supplementary file 3 — Table S2. The number of missing tadpoles for each of three host species Anaxyrus boreas (A), Pseudacris regilla (P), and Rana cascadae (R) across 7 species combinations and two pathogen treatments (Bd). [file ece30005-1432-sd3.docx]

| **Table S2** |  |  |  |  |  |  |  |  |
| --- | --- | --- | --- | --- | --- | --- | --- | --- |
|  |  |  |  |  |  |  |  |  |
| Focal spp |  | B | | P | | R | | Totals |
| Bd treatmt |  | Bd+ | Bd- | Bd+ | Bd- | Bd+ | Bd- |  |
| Spp Combo | B | 0 | 1 | 0 | 0 | 0 | 0 | 1 |
|  | BR | 10 | 11 | 0 | 0 | 2 | 5 | 28 |
|  | P | 0 | 0 | 4 | 0 | 0 | 0 | 4 |
|  | PB | 0 | 1 | 1 | 0 | 0 | 0 | 2 |
|  | PBR | 8 | 5 | 5 | 4 | 0 | 2 | 24 |
|  | PR | 0 | 0 | 10 | 9 | 2 | 3 | 24 |
|  | R | 0 | 0 | 0 | 0 | 9 | 13 | 22 |
| Totals |  | 18 | 18 | 20 | 13 | 13 | 23 | 105 |
