# Supplementary material for: Host species composition influences infection severity among amphibians in the absence of spillover transmission
Source: Ecol Evol. 2015 Mar 5;5(7):1432–9. doi: 10.1002/ece3.1385 (PMC4395173; doi:10.1002/ece3.1385)
Supplement: Supplementary file 4 — Table S3. Generalized linear models for mean growth in mass (mg) and length (mm) in tadpoles of two amphibian host species among four species combinations (Combo) and two pathogen treatments (Bd). Models include a two-way interaction term (Bd*combo) and Day as a covariate. [file ece30005-1432-sd4.docx]

| **Table S3** |  |  |  |  |  |  |  |  |
| --- | --- | --- | --- | --- | --- | --- | --- | --- |
|  | *Pseudacris* | | | | *Rana* | | | |
| Mass: | DF | MS | F | *p* | DF | MS | F | *p* |
| Bd | 1 | 63498.61 | 1.024 | 0.32 | 1 | 572516.29 | 5.77 | **0.02** |
| Combination | 3 | 60924.03 | 0.98 | 0.41 | 3 | 45560.17 | 0.46 | 0.71 |
| Bd*Combo | 3 | 88746.77 | 1.43 | 0.25 | 3 | 182200.53 | 1.84 | 0.15 |
| Day | 1 | 104567.83 | 1.69 | 0.2 | 1 | 260266.81 | 2.625 | 0.11 |
| Error | 48 | 61990.08 |  |  | 51 | 99165.94 |  |  |
| Length: |  |  |  |  |  |  |  |  |
| Bd | 1 | 43.78 | 4.5 | **0.039** | 1 | 150.83 | 13.49 | **0.001** |
| Combination | 3 | 4.09 | 0.42 | **0.023** | 3 | 3.43 | 0.31 | 0.82 |
| Bd*Combo | 3 | 8.74 | 0.9 | 0.45 | 3 | 31.9 | 2.85 | **0.04** |
| Day | 1 | 0.13 | 0.014 | 0.9 | 1 | 16.16 | 1.45 | 0.24 |
| Error | 48 | 9.74 |  |  | 51 | 11.18 |  |  |
