# Supplementary material for: Host species composition influences infection severity among amphibians in the absence of spillover transmission
Source: Ecol Evol. 2015 Mar 5;5(7):1432–9. doi: 10.1002/ece3.1385 (PMC4395173; doi:10.1002/ece3.1385)
Supplement: Supplementary file 5 — Appendix S1. Materials and methods. [file ece30005-1432-sd5.doc]

**Supplementary Materials and Methods**

*Animal husbandry.* Amphibian eggs were collected near Todd Lake (44° 1' 48", -121° 41' 23.9994", 1873 m above sea level) from the Oregon Cascades Range (Deschutes County, OR): *Rana cascadae* (*Rana*: 5 clutches), *Anaxyrus boreas* (*Anaxyrus:* 5 partial clutches), and *Pseudacris regilla* (*Pseudacris*: 25 partial clutches). Eggs were reared in the laboratory (14-15ºC) at Oregon State University in 9 L aquaria with dechlorinated and aerated water until hatching. Upon hatching, conspecific tadpoles from various clutches were combined at densities of 150 tadpoles per aquarium. Tadpoles were moved into experimental groups and inoculated with Bd or a control treatment at the free-swimming stage (stage 25-27, [1]) within experimental containers (30 x 17 x 9 cm) filled with 2.5 L filtered water.

*Inoculation with* Batrachochytrium *(Bd).* Fifty-six culture dishes of *Batrachochytrium dendrobatidis* JEL strain 274 on 1% Tryptone agar were flooded with 5 mL filtered water and gently scraped with a rubber policeman three times across the diameter of the plate to dislodge colonies. The liquid Bd suspension from each culture dish was combined to yield a total zoospore concentration of approximately 1.46 x 10^8^ zoospores/mL. The same method was used to obtain control inoculum using sterile plates (1% Tryptone agar) without Bd. Experimental units were inoculated once at the beginning of the 40-day experimental period with 3 mL of either Bd inoculum (Bd^+^ treatment) or control agar wash (Bd^-^ treatment). Animals were fed a 2:1 mixture of ground alfalfa pellets and fish flakes (Tetramin) throughout the 40-day period (one pinch per container, approximately 0.22 g) when it was observed that no more food remained on the surface of the water (i.e., when food was completely consumed). Containers were checked daily for mortality. Many amphibian species exhibit opportunistic cannibalism during the larval stage (*pers. obs.*, [2]). Thus, if a tadpole was missing from a container it was assumed to have been consumed by another tadpole.

*Quantifying infection using real-time PCR.* Mouthparts were excised from all Bd^+^ and a subsample of Bd^-^ tadpoles (N=324 animals from 56 test chambers) for quantitative real-time PCR analysis. DNA was extracted from tissue using a DNAeasy 96 well kit (Qiagen, Valencia, California) and quantified using a spectrophotometer (Nanodrop Technologies, Wilmington, Delaware). Real-time PCR was based on established methodology [3] using an ABI 7300 Real-time PCR system (Applied Biosystems, Foster City, California). Twenty five µL reactions contained 5µL of 20ng/L template DNA and 20µL of master mix (containing 900nM forward and reverse primers, 125nM MGB probe, and Taqman Master Mix). We obtained Bd genome equivalent standards from D. Boyle (as used in [3]) and included triplicates of each standard serially diluted on each plate (10^-1^, 10^0^, 10^1^, 10^2^) and a duplicate of the high standard (10^3^). Unknown samples were run in triplicate and values that differed by a coefficient of variation greater than 0.2 were rerun for greater accuracy. This measure accounts for differences in Bd infection severity between species based on size alone (i.e., more mouthpart tissue containing more zoospores).

**References**

1. Gosner KL. A simplified table for staging anuran embryos and larvae with notes on identification. Herpetologica. 1960;16:183-90.

2. Crump ML. Opportunistic cannibalism by amphibian larvae in temporary aquatic environments. Am Nat. 1983;121(2):281-9.

3. Boyle DG, Boyle DB, Olsen V, Morgan JAT, Hyatt AD. Rapid quantitative detection of chytridiomycosis (*Batrachochytrium dendrobatidis*) in amphibian samples using real-time Taqman PCR assay. Dis Aquat Org. 2004;60:141-8.
